# First person – Michel A. K. Dongmo

**DOI:** 10.1242/bio.058717

**Published:** 2021-04-15

## Abstract

First Person is a series of interviews with the first authors of a selection of papers published in Biology Open, helping early-career researchers promote themselves alongside their papers. Michel Dongmo is first author on ‘Local adaptation in thermal tolerance for a tropical butterfly across ecotone and rainforest habitats’, published in BiO. Michel conducted the research described in this article while a Research Assistant in Drs Rachid Hanna and Komi Fiaboe's lab at the International Institute of Tropical Agriculture, Yaoundé, Cameroon. Michel is now a postdoc in the lab of Dr Timothy Bonebrake at the School of Biological Sciences, The University of Hong Kong, China, investigating the response and adaptation of insect populations to ongoing climate change in the field of agriculture and conservation biology in Central Africa.


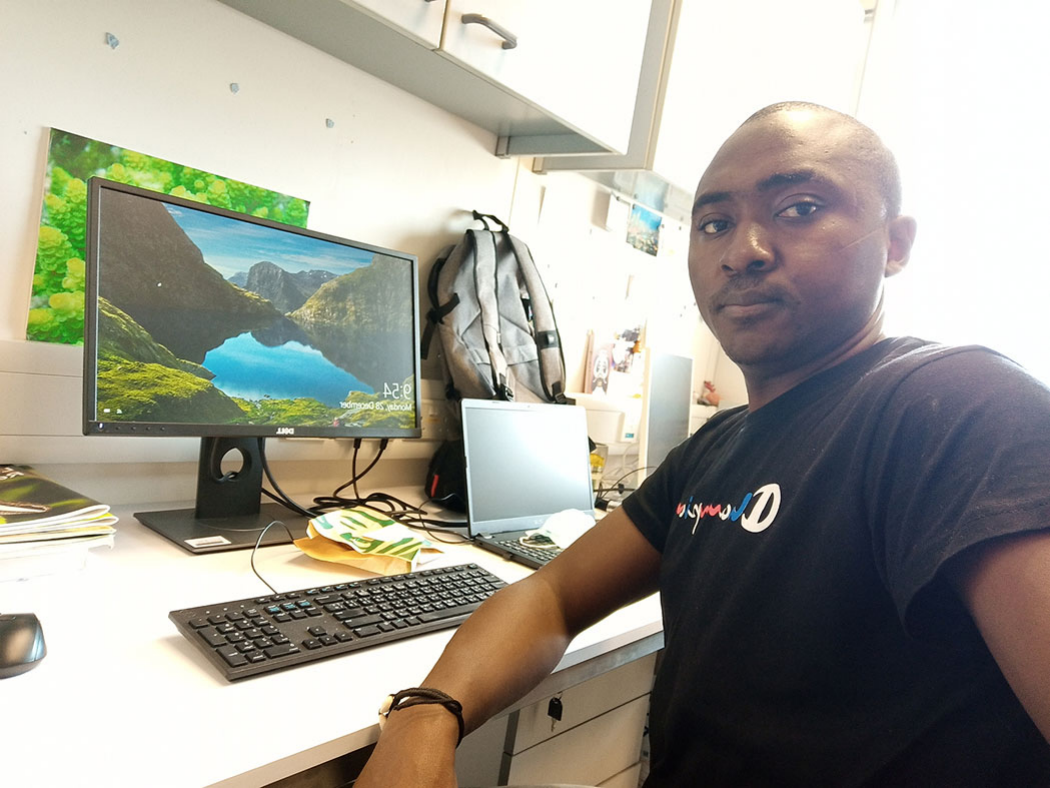


**Michel A. K. Dongmo**

**What is your scientific background and the general focus of your lab?**

My scientific background is in thermal biology applied to crop protection and conservation biology. I was trained by Dr Rachid Hanna as an entomologist when I was doing my Master's degree at the International Institute of Tropical Agriculture and the University of Yaoundé, Cameroon. The goal of my Master's thesis was to model the effect of different thermal regimes on some life history of the oriental fruit fly *Bactrocera dorsalis*, which causes tremendous losses to fruit and vegetable production in many parts of the world. I did the same work with colleagues on other pests such as the banana aphid (*Aphis gossypii*) and on the fruit fly parasitoid *Fopius arisanus*. Later on, Dr Timothy Bonebrake became the main supervisor for my PhD thesis where I investigated the morphological variation and the thermal stress response of different populations of the butterfly species *Bicyclus dorothea* on a forest/ecotone gradient. After my PhD defence, I took up a postdoctoral position in Dr Timothy's laboratory at the University of Hong Kong. Here we are using Mycalesine butterflies – genus Bicyclus – to assess the role of plasticity in buffering thermal stress response in a forest and ecotone gradient in Cameroon. Generally, Dr Timothy's lab focuses on how current climate variation affects the physiology, redistribution and some evolutionary aspects of animal species.

**How would you explain the main findings of your paper to non-scientific family and friends?**

Tropical Africa is the home of some of the most important biodiversity on earth and, unfortunately, this biodiversity is facing threats from recent global warming. In Cameroon, there is an important variation regarding habitat type in which we can find different populations of a given species. The main findings of our research indicate that populations of the butterfly *B. dorothea* inhabiting ecotones (which are particular landscapes made of transitional zones between two types of ecosystems, forest and savannah in our case) are more likely to cope with thermal stress under laboratory conditions compared to population inhabiting rainforests. In fact, climate components such as temperature vary in these habitats, leading to a kind of adaptation of cool-blooded animals inhabiting them. Hence, insects found in habitats with more temperature variation will likely be able to withstand high temperatures compared to those living in habitats with low temperature variation. The direct consequence of this is that warming temperatures can lead to the vulnerability of insects found in stable habitats such as rainforests.

“…insects found in habitats with more temperature variation will likely be able to withstand high temperatures compared to those living in habitats with low temperature variation.”

**What are the potential implications of these results for your field of research?**

The findings of our research show two important points for climate mitigation in the field of conservation biology: 1) thermally variable landscapes likely contribute to driving local adaptation of some animal populations to harsh climate variation, but questions remain to what extend this can occur; 2) thermally stable habitats such as dense tropical forests harbour species that are more likely to be vulnerable to climate variation and habitat fragmentation. Few studies have been carried out on the effect of global warming on the physiology of animals in Central Africa. This research is then an important contribution that will help to understand how climate change affects ectotherms differently regarding the type of their habitat, and is a key tool in forming policies of ecosystem management.

**What has surprised you the most while conducting your research?**

Even though differences in thermal tolerance were expected at the beginning of the study, I was surprised by the clear differences observed in thermal tolerance tested on the second-generation individuals originating from distinct habitats. This indicates that there is a potential evolutionary phenomenon going on within this species related to climate adaptation. Regarding the complexity and the diversity of natural habitats that can be found in Cameroon, each habitat is likely to shape the thermal breadth of ectotherms that can be found there!

**Figure:**
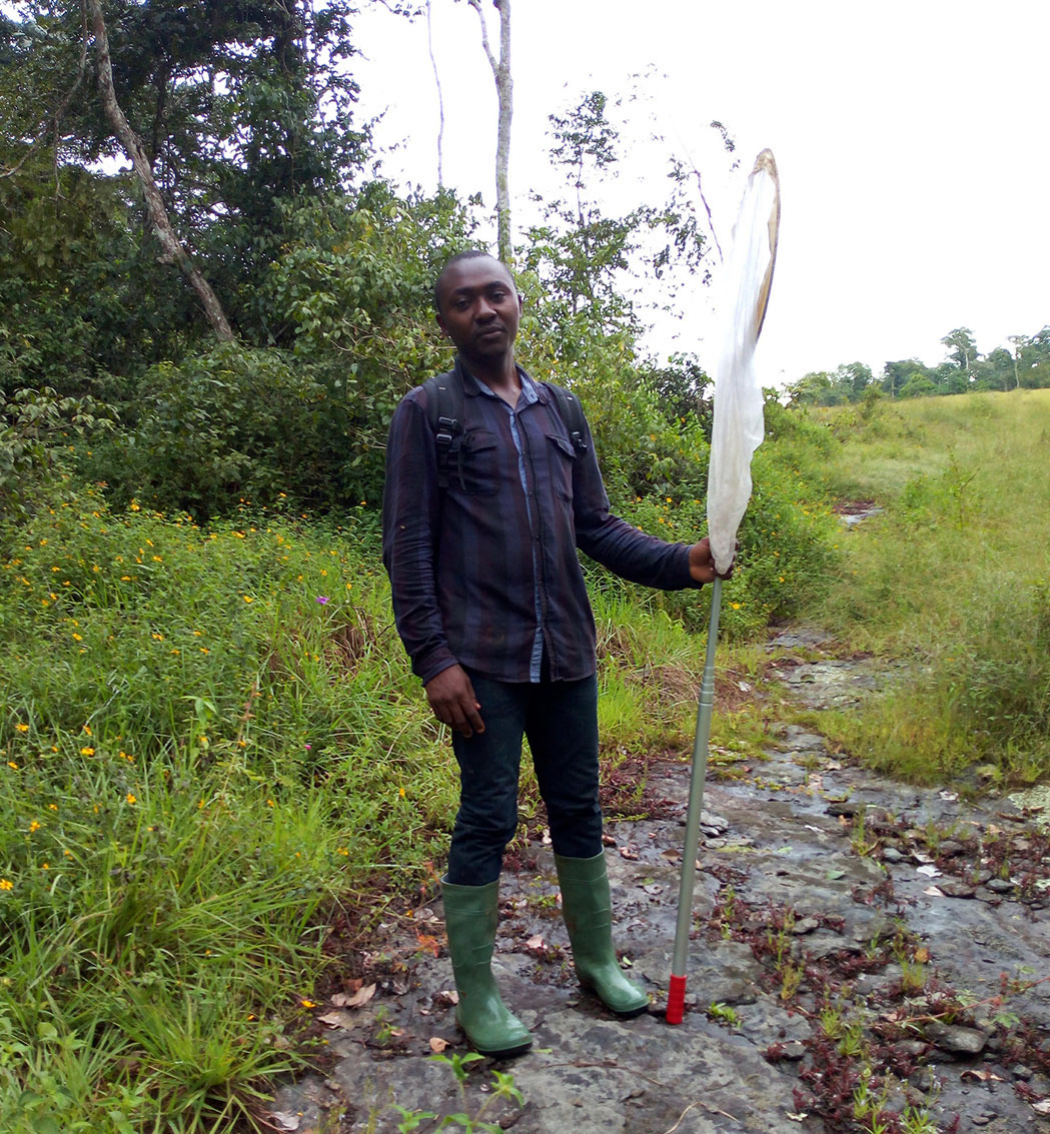
**Butterfly monitoring at the Dja Faunal Reserve in Cameroon.**

**What changes do you think could improve the professional lives of early-career scientists?**

After they have defended their PhD, early-career scientists most of the time have good project ideas but their limitations remain in gaining access to funding to conduct those projects. Besides that, getting a permanent position nowadays is becoming more and more difficult. So, to my view, access to funding is important and also collaboration between well-established scientists and early career scientists must be encouraged and developed.

**What's next for you?**

The next challenge for me is to explore the genetic implication in the response of ectotherms to recent climate change in Central Africa.
